# Metabolic interrogation as a tool to optimize chemotherapeutic regimens

**DOI:** 10.18632/oncotarget.15186

**Published:** 2017-02-08

**Authors:** Vlad C. Sandulache, Yunyun Chen, Lei Feng, William N. William, Heath D. Skinner, Jeffrey N. Myers, Raymond E. Meyn, Jinzhong Li, Ainiwaer Mijiti, James A. Bankson, Clifton D. Fuller, Marina Y. Konopleva, Stephen Y. Lai

**Affiliations:** ^1^ Department of Head and Neck Surgery, The University of Texas MD Anderson Cancer Center, Houston, Texas, USA; ^2^ Bobby R. Alford Department of Otolaryngology Head and Neck Surgery, Baylor College of Medicine, Houston, TX, USA; ^3^ Department of Biostatistics, The University of Texas MD Anderson Cancer Center, Houston, Texas, USA; ^4^ Department of Thoracic/Head and Neck Medical Oncology, The University of Texas MD Anderson Cancer Center, Houston, Texas, USA; ^5^ Department of Radiation Oncology, The University of Texas MD Anderson Cancer Center, Houston, Texas, USA; ^6^ Department of Experimental Radiation Oncology, The University of Texas MD Anderson Cancer Center, Houston, Texas, USA; ^7^ Department of Oral and Maxillofacial-Head and Neck Surgery, Beijing Stomatological Hospital, Beijing, China; ^8^ Department of Oral and Maxillofacial Surgery, The First Affiliated Hospital of Xinjiang Medical University, Xinjiang, China; ^9^ Department of Imaging Physics, The University of Texas MD Anderson Cancer Center, Houston, Texas, USA; ^10^ Department of Leukemia, The University of Texas MD Anderson Cancer Center, Houston, Texas, USA; ^11^ Department of Molecular and Cellular Oncology, The University of Texas MD Anderson Cancer Center, Houston, Texas, USA

**Keywords:** cisplatin, p53, lactate, head and neck squamous cell carcinoma, acute myelogenous leukemia

## Abstract

Platinum-based (Pt) chemotherapy is broadly utilized in the treatment of cancer. Development of more effective, personalized treatment strategies require identification of novel biomarkers of treatment response. Since Pt compounds are inactivated through cellular metabolic activity, we hypothesized that metabolic interrogation can predict the effectiveness of Pt chemotherapy in a pre-clinical model of head and neck squamous cell carcinoma (HNSCC).

We tested the effects of cisplatin (CDDP) and carboplatin (CBP) on DNA damage, activation of cellular death cascades and tumor cell metabolism, specifically lactate production. Pt compounds induced an acute dose-dependent, transient drop in lactate generation *in vitro*, which correlated with effects on DNA damage and cell death. Neutralization of free radical stress abrogated these effects. The magnitude of this effect on lactate production correlated with the differential sensitivity of HNSCC cells to Pt compounds (CDDP vs CBP) and p53-driven Pt chemotherapy resistance. Using dual flank xenograft tumors, we demonstrated that Pt-driven effects on lactate levels correlate with effects on tumor growth delay in a dose-dependent manner and that lactate levels can define the temporal profile of Pt chemotherapy-induced metabolic stress. Lactate interrogation also predicted doxorubicin effects on cell death in both solid tumor (HNSCC) and acute myelogenous leukemia (AML) cell lines.

Real-time metabolic interrogation of acute changes in cell and tumor lactate levels reflects chemotherapy effects on DNA damage, cell death and tumor growth delay. We have identified a real-time biomarker of chemotherapy effectiveness which can be used to develop adaptive, iterative and personalized treatment regimens against a variety of solid and hematopoietic malignancies.

## INTRODUCTION

Platinum-based (Pt) chemotherapy is broadly employed in the treatment of solid tumors and is incorporated into multi-drug regimens for hematopoietic malignancies [1–6]. Its anti-tumor effectiveness is primarily driven by the extent of induced DNA damage, and activation of cellular death cascades [7–9]. Changes in drug design, delivery and dosing over the last few decades have been used to improve anti-tumoral activity, while improving the side effect profile which can be substantial for most current clinical regimens [10–16].

Although Pt agents can achieve significant anti-tumor activity alone or in combination with other agents and ionizing radiation (IR), resistance has been described in both solid and hematopoietic tumors [11]. *In vitro* studies have identified multiple mechanisms of Pt therapy resistance and preclinical and clinical studies have identified genomic (i.e. *TP53* mutation) and epigenetic events which are associated with decreased tumor response [17]. While preclinical studies can be used to explain resistance and tissue analysis can be used to identify markers associated with response or resistance at the population level, predicting individual tumor response to Pt treatment remains a challenge.

The effectiveness of Pt treatment is influenced by the metabolic and energetic state of tumor cells [7, 18–23]. Neutralization of Pt compounds by reducing proteins such as glutathione can prevent entry into the nucleus and generation of DNA damage [21, 23–26]. Regeneration of these reducing proteins requires cellular expenditure of energy as well as secondary reducing equivalents (i.e. NADH/NADPH). We previously showed that ionizing radiation-induced ROS trigger temporary perturbations in cellular and tumor reducing potential [27, 28]. This results in a secondary alteration of the rate of conversion of pyruvate into lactate due to depletion of the reducing equivalent pool [27, 28]. Interrogation of cell and tumor lactate levels can therefore provide real-time information regarding treatment effect. Based on these data, we hypothesized that lactate-based metabolic interrogation can predict the effectiveness of Pt regimens. To confirm translational relevance we aimed to demonstrate the following: 1) dose-dependent relationship between acute, transient changes in lactate levels and Pt agent dose; 2) correlation with intrinsic (*TP53* mutation driven) Pt treatment resistance; and 3) generalizability across multiple histologies and multiple Pt agents of varying cytotoxicity. Finally, to determine whether our findings can be generalized to other chemotherapeutic agents, we sought to confirm the validity of our mechanism using doxorubicin, a drug commonly utilized in the treatment of both solid tumors and leukemias [29–33].

## RESULTS

### Cisplatin induces cell cycle arrest and cell death

**Figure 1:**
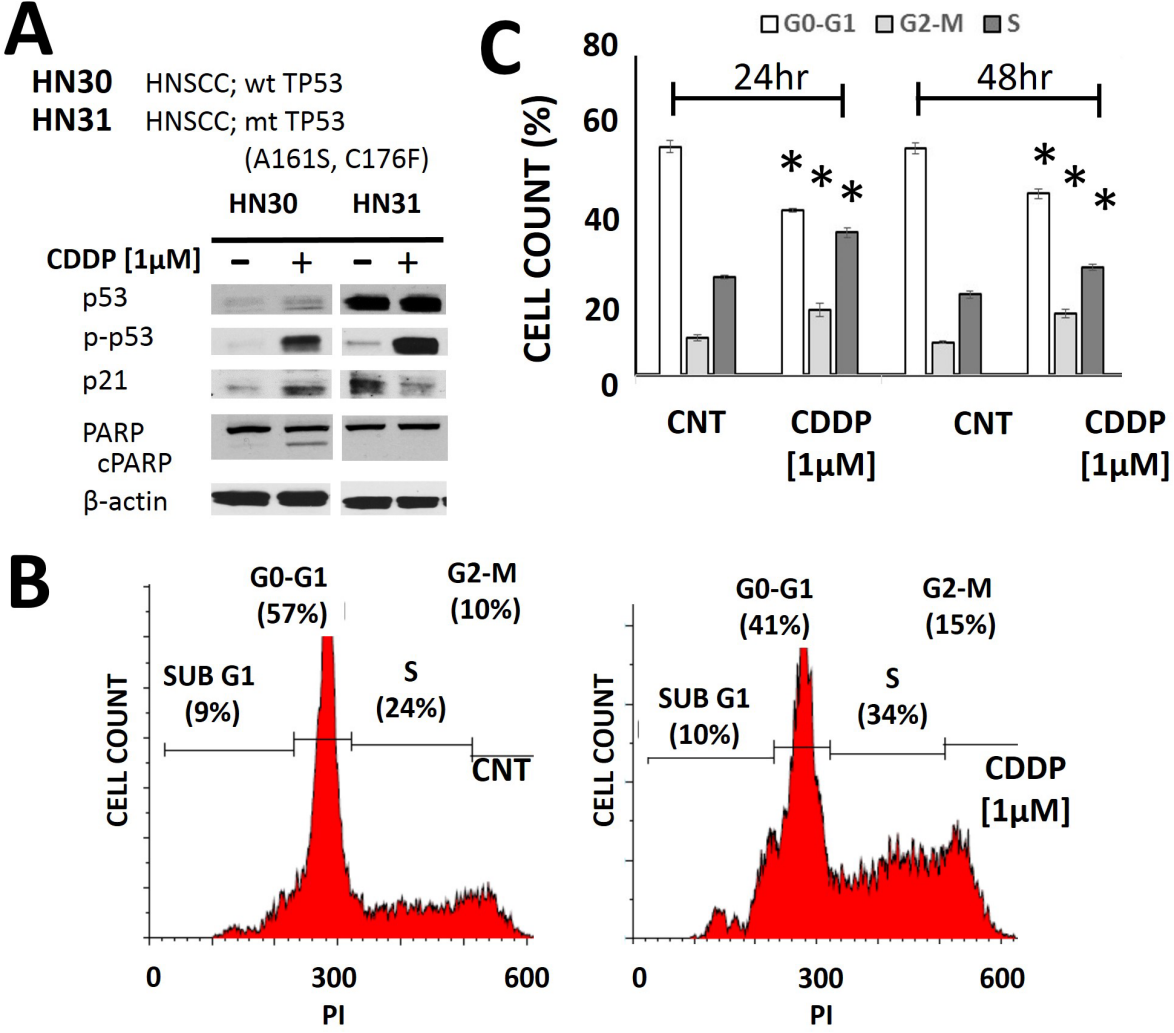
Cisplatin activates p53 and induces cell cycle arrest **A**. HNSCC cells (HN30 wild type (wt) *TP53* and HN31 mutant (mt) *TP53*) were exposed to CDDP for 24hr and protein lysates were analyzed via Western Blotting. **B-C**. Cells (HN30) were exposed to CDDP [1μM] and harvested at 24hr (B- representative panels, C- summary of data) or 48hr post exposure. Effects on cell cycle were ascertained using propidium iodine staining. CDDP induced an increase in cellular G2-M and S phase. Each experiment was carried out at least in triplicate. * indicates p<0.05 compared to the control condition.

Two parental human HNSCC cell lines were used: HN30 and HN31. These cell lines have been previously described in detail by our group. Briefly, the cell lines are derived from the primary tumor and a metastatic site of a HNSCC patient and are isogenic with the exception of the mutational status of *TP53* (Figure 1) [34, 35, 39–41]. Treatment of HNSCC cells with CDDP resulted in phosphorylation and stabilization of p53 along with increased levels of p21 and PARP cleavage in HN30 cells which express wild type *TP53* (Figure 1A). As expected, the extent of PARP cleavage was decreased in HN31 which is consistent with its relative cisplatin resistance. In our experience, the HNSCC cell lines including those used in this study do not exhibit significant rates of apoptosis in response to genotoxic stress [41]. Consistent with this experience, CDDP exposure primarily triggered cell cycle arrest, as demonstrated by the increased cellular fractions in G2-M and S phase (Figure 1B-1C).

### Cisplatin decreases cellular lactate levels

**Figure 2:**
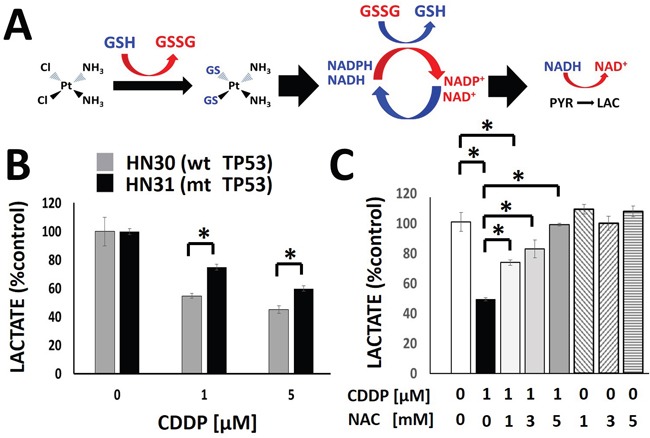
Cisplatin triggers a rapid, concentration dependent decrease in cellular lactate production **A**. Model of the hypothesized effects of CDDP on lactate production. The chloride (Cl) moieties are replaced upon exposure to glutathione (GSH), inactivating CDDP. Regeneration of GSH utilizes secondary reducing equivalents (NAD(P)H) and decreases the conversion of pyruvate into lactate. **B**. CDDP induced a concentration-dependent decrease in cellular lactate levels at 1hr following drug exposure. HN30 cells demonstrated approximately 25% greater decrease in lactate levels compared to their mutant *TP53* counterpart, HN31, at each concentration. **C**. HN30 were exposed to CDDP [1μM] in the presence or absence of N-acetyl cysteine (NAC) [1-5mM]. Lactate levels were measured at 1hr following drug exposure. * indicates p-value < 0.05 compared to corresponding control condition. All values were normalized to corresponding control condition. Each experiment was carried out at least in triplicate, with values indicating means and error bars representing standard deviation.

CDDP can be inactivated by primary reducing equivalents (i.e. glutathione) which are regenerated via utilization of secondary reducing equivalents [21, 23, 24, 26]. As previously shown by our group using radiation-induced free radical stress, acute perturbations in levels of reducing equivalents can be reflected in an altered rate of pyruvate to lactate conversion (Figure 2A) [27, 28]. Exposure to CDDP triggered a rapid (1hr), concentration-dependent decrease in cellular lactate levels (Figure 2B). Treatment with CDDP is associated with approximately 25% more pronounced decrease in HN30 cellular lactate levels as compared to HN31. This difference is consistent with the greater sensitivity of HN30 to CDDP compared to HN31 measured using both MTT assay (GI50 HN30-1.94μM, HN31- 2.99μM) and clonogenic survival assay (Supplementary Figure 1). The addition of the ROS scavenger NAC reversed the decrease in lactate levels triggered by CDDP exposure in a dose-dependent fashion, consistent with a ROS-mediated mechanism (Figure 2C). NAC reversal of CDDP-induced lactate changes correlated with NAC reversal of CDDP toxicity as measured using clonogenic assays survival (Supplementary Figure 1). This is indicative of a shared ROS-mediated mechanism for both effects.

In order to determine whether changes in enzyme levels may be responsible for the measured decrease in lactate following CDDP administration, we evaluated LDHa and LDHa protein levels by Western blot and noted no change within the time frame for acute lactate perturbations (Supplementary Figure 2). To evaluate the potential validity of the model proposed in Figure 2, we measured NADH/NAD+ and NADPH/NADP+ levels and ratios under conditions of cisplatin exposure and found a good correlation between changes in the NADH/NAD+ ratio and cisplatin induced changes in intra-cellular lactate levels. The relative time frame, dose dependence and p53-dependence was consistent between changes in NADH/NAD+ and lactate (Figure 2, Supplementary Figure 2). NADPH/NADP+ levels were also perturbed, though to a smaller degree. (Supplementary Figure 2C).

To evaluate the duration of CDDP-induced lactate decreases, we performed a washout experiment (Supplementary Figure 3A). Following administration of CDDP at 1μM for 1hr, the drug was withdrawn and the media replaced with fresh media. Cellular lactate levels began to demonstrate recovery at approximately 6 hours following CDDP removal and returned to near baseline levels by 24 hours following drug withdrawal. Cell viability was measured using MTT at all time points to insure that the acute changes in lactate levels are not due to a decrease in cell number secondary to acute CDDP toxicity (Supplementary Figure 3B). These data mirror those we previously described with radiation induced lactate changes, although with slower overall temporal kinetics [27, 28].

### Lactate levels reflects differential cisplatin sensitivity driven by p53 status

Cells without functional p53 (HN31) exhibited a reduced decrease in lactate following CDDP exposure, as compared to HN30 cells (Figure 2). We have previously demonstrated that the metabolic impact of *TP53* mutation in this cell line background is predominantly driven by a loss of function mechanism and is reproduced by knockdown of functional p53 in the HN30 cell line [34, 35, 39–41]. To confirm that the impact of *TP53* mutation on lactate levels is in fact due to loss of p53 function, we repeated the experiment in a previously described cell line pair: HN30lenti and HN30shp53. Consistent with a p53-driven mechanism, CDDP induced a reduced decrease in cellular lactate in cells lacking functional p53 (HN30shp53) compared to lentiviral infected control cells (HN30lenti) (Supplementary Figure 4). These data are consistent with recently published data which demonstrate a similar phenomenon in the HN30lenti HN30shp53 cell lines using radiation [28].

### Acute, transient lactate changes reflect cisplatin effects on tumor growth delay

**Figure 3:**
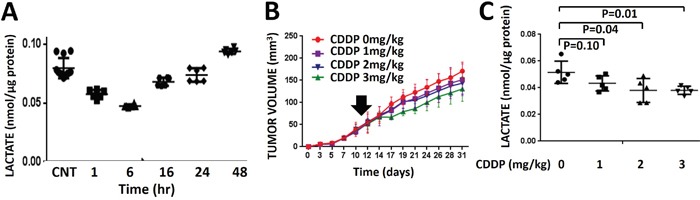
Cisplatin-induced lactate perturbations are transient and correlate with effects on growth delay **A**. Dual HNSCC (HN31) flank tumors were exposed to a single dose of CDDP (3mg/kg). Tumors were harvested at various time points (n=2) following CDDP administration and lactate levels were measured biochemically and compared to untreated control tumors (n=4). Each tumor was analyzed in triplicate. **B**. Dual HNSCC (HN31) flank tumors were exposed to a single dose of CDDP administered at day 11. One tumor (n=5/dose) was allowed to grow. Measurements of tumor size were obtained for the remainder of the experimental period and correlated to post-exposure lactate levels. Data are presented as mean volume with error bars indicating standard deviation. **C**. One tumor per animal (n=5/dose) was removed and analyzed biochemically for lactate levels at 3 hr post-exposure.

We have previously shown that radiation triggers a transient decrease in intra-tumoral lactate levels [27, 28]. Consistent with these data, we detected a transient decrease in tumor lactate levels following administration of CDDP *in vivo*. Following administration of a single dose of CDDP to mice bearing dual flank tumors, tumor tissue was harvested and analyzed biochemically for lactate levels at various time points following drug administration. Lactate levels reached nadir at 6 hours post-CDDP exposure and recovered to baseline, untreated levels between 24-48 hours post-exposure (Figure 3A). Overall the kinetics of CDDP-induced lactate changes were noted to be slower than those previously measured in response to radiation (data not shown). HNSCC (HN31) flank tumors were allowed to grow for 11 days prior to administration of a single dose of CDDP. One tumor was removed and lactate levels were measured at 3 hr post treatment, while the remaining tumor was allowed to grow. These tumor lactate levels measured immediately following CDDP administration correlated with the dose-dependent delay in tumor growth recorded over the subsequent 3 week time period (Figure 3B-3C). These effects were reproduced at higher CDDP doses with similar effects (Supplementary Figure 5) The CDDP-resistant cell line was used for these experiments for two reasons. First, we wanted to demonstrate that lactate levels reflect small, subtle effects on tumor growth delay and not simply large dramatic changes in dose response as would be expected with a CDDP sensitive cell line. Second, HN31 demonstrates an aggressive tumor growth pattern consistent with the clinical behavior exhibited by HNSCC in human patients.

### Metabolic interrogation can differentiate between carboplatin and cisplatin effects

**Figure 4:**
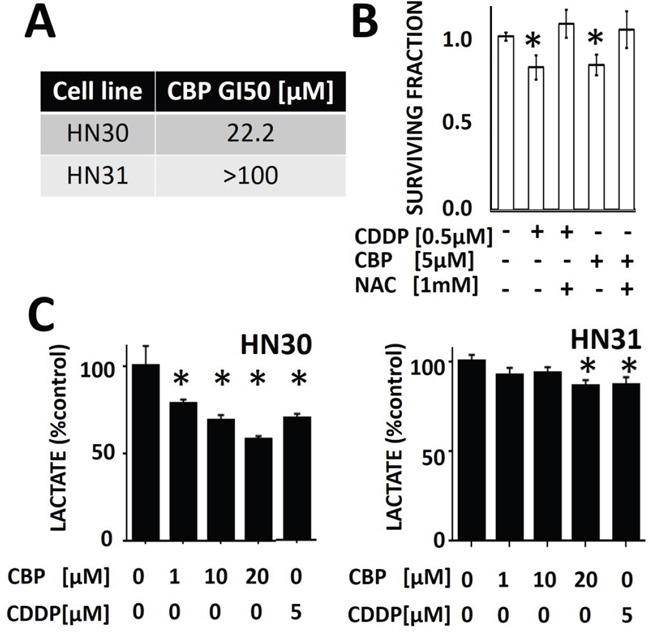
Differential sensitivity of HNSCC cells to cisplatin (CDDP) and carboplatin (CBP) **A**. Using standard MTT assay, GI50 values were calculated for both HN30 and HN31. **B**. HNSCC (HN31) cells were exposed to CBP or CDDP in the presence or absence of NAC. Differential effects on survival were calculated using clonogenic survival assay. **C**. HNSCC cells were exposed to CBP or CDDP for 1hr prior to measurements of cellular lactate levels. HN30 cells demonstrated approximately 25-60% decrease in lactate levels across the range of tested CBP doses compared to approximately 10% decrease in their counterpart, HN31. * indicates p-value < 0.05 compared to corresponding control condition. All values normalized to corresponding control condition. Each experiment was carried out at least in triplicate, with values indicating means and error bars representing standard deviation.

Carboplatin (CBP) is employed in the treatment of HNSCC when patients cannot tolerate the side effect profile of CDDP. In our preclinical model, HNSCC cells are less sensitive to CBP compared to CDDP (HN30 CBP GI50 [22.2μM] vs CDDP GI50 [1.94μM]; HN31 CBP GI50 [>100μM] vs CDDP GI50 [2.99μM] (Figure 4A); importantly, HN30 cells are more sensitive to both CBP and CDDP compared to HN31 cells. The differential GI50 for CBP and CDDP was confirmed using a clonogenic assay which demonstrates that a substantially higher CBP concentration is required to achieve a decrease in surviving fraction comparable to CDDP ([5μM] vs [0.5μM] respectively) (Figure 4B). The differential sensitivity of HNSCC cells (both HN30 and HN31) for CBP compared to CDDP is reflected in acute lactate perturbations following treatment with the two compounds. Specifically, CBP requires a higher concentration to induce the same decrease in cellular lactate levels compared to CDDP (Figure 4C). The effects of both CDDP and CBP on lactate levels are blunted in the absence of functional p53 (HN31) (Figure 4C). The differential sensitivity to CBP and CDDP with respect to lactate perturbations was intensified at time points greater than 1 hr (Supplementary Figure 6).

### Cisplatin effects on p53 activation and DNA damage are dependent on cellular ROS

**Figure 5:**
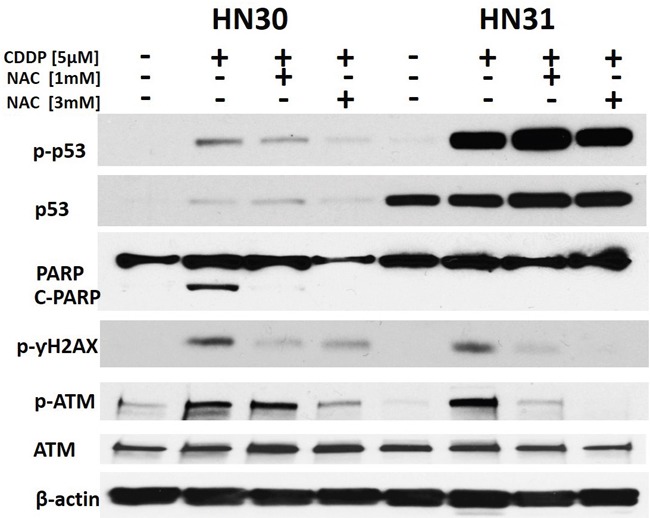
Cisplatin effects on p53 phosphorylation and DNA damage are reversed by a free radical scavenger HN30 and HN31 cells were exposed to CDDP in the presence or absence of N-acetyl cysteine (NAC). 0194H2X and ATM phosphorylation was ascertained as a measure of DNA damage along with activation of p53 (phospho) and cleavage of PARP. Total p53 levels are high and stable in HN31 consistent with the mutational status of *TP53* in this cell line. PARP cleavage and 0194H2X phosphorylation is reduced in HN31 compared to HN30.

HN30 cells were exposed to CDDP and activation of DNA damage signaling pathways was ascertained. CDDP triggers γH2X and ATM phosphorylation levels consistent with its putative mechanism of action. In the presence of NAC (a scavenger of reactive oxygen species (ROS)), these effects are blunted (Figure 5). Overall, CDDP effects are blunted in HN31 cells consistent with their relative CDDP resistance.

### Cisplatin effects on acute lactate levels are reproducible across tumor histologies

Anaplastic thyroid carcinoma (ATC) cells (U-HTH-83) demonstrated a dose-dependent decrease in cellular lactate levels following CDDP administration (Supplementary Figure 7A). When CBP is substituted for CDDP, a higher dose (CBP [20μM] vs CDDP [5μM]) increase is required to achieve the same effect (Supplementary Figure 7B).

### Lactate levels are predictive of doxorubicin effects in both HNSCC and AML

**Figure 6:**
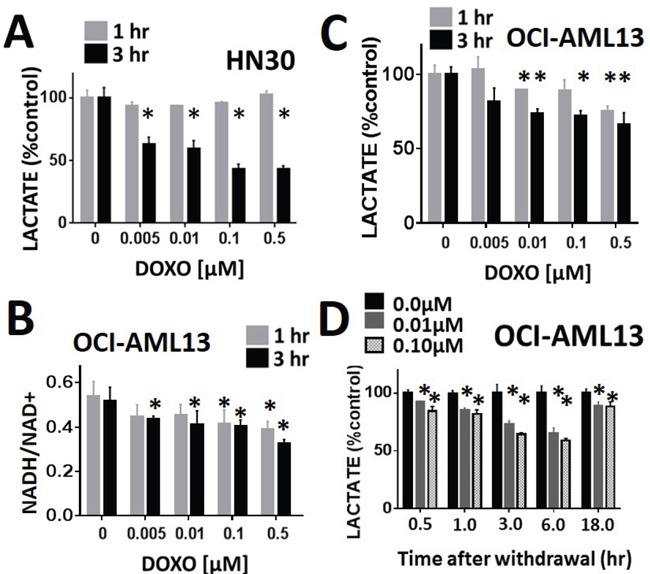
Lactate interrogation predicts doxorubicin cytotoxic effects in HNSCC and ALL Doxorubicin induced a dose-dependent decrease in cellular lactate levels in HNSCC HN30 **A**. and AML OCI-AML13 **C**. cell lines. **B**. OCI-AML13 cells were exposed to increasing doses of doxorubicin. NADH and NAD+ levels were ascertained biochemically and the ratio was calculated. Doxorubicin induced a dose dependent decrease in the NADH/NAD+ ratio. **D**. AML cells were exposed to either control media or media with doxorubicin at 0.01 and 0.1 μM for 1hr. Doxorubicin was then removed and cells were harvested at various time points following withdrawal. Lactate levels were measured at each time point and compared to the control (untreated) condition. * indicates p-value < 0.05 compared to corresponding control condition. All values normalized to corresponding control condition. Each experiment was carried out at least in triplicate, with values indicating means and error bars representing standard deviation.

The effects of doxorubicin on cellular lactate levels were ascertained using both HNSCC (HN30) and AML (OCI-AML3) cell lines. In both cell lines, doxorubicin induced a dose-dependent decrease in cellular lactate levels acutely following exposure (Figure 6A and 6C); this effect occurred close to the GI50 of both cell lines: HN30 (0.01μM) and OCI-AML13 (0.01μM). As previously shown in HNSCC cells, the acute decrease in lactate mirrored an acute reduction in the NADH/NAD+ ration, consistent with a free radical mediated effect (Figure 6B). Doxorubicin effects on AML lactate levels were demonstrated to be transient, resulting in recovery of lactate levels following drug withdrawal Figure 6D.

## DISCUSSION

Personalization of chemotherapy regimens is essential to improving clinical outcomes while maintaining acceptable toxicity. Realizing this clinical goal requires three steps. First, drug chemistry must be sufficiently advanced to allow for agent substitution within the same family of compounds. Second, the potential for secondary agent modification to adjust drug kinetics and optimize preferential tumor uptake must be realized. Third, development of predictive biomarkers of actual drug response must be completed to allow for individualized therapeutic regimens. Platinum chemistry has been continuously refined over the last few decades resulting in multiple, clinically-approved derivatives with variable toxicity profiles and pharmacokinetics [10, 14, 42]. The continued development of newer agents is likely to further increase the availability of Pt agents for improved therapeutic regimens [43, 44]. The development of suitable biomarkers of Pt compound response, combined with these improvements in compound chemistry is likely to generate truly personalized treatment regimens.

Over the last decade, increasing efforts to characterize the genomic, epigenetic and molecular profile of solid and hematopoietic tumors have provided an increased understanding of tumor biology and treatment response [45, 46]. Several markers of treatment (i.e. CDDP, radiation) resistance in head and neck squamous cell carcinoma (HNSCC) have been described in the literature [17, 41, 47]. Unfortunately, because many of these markers are either very specific to a single treatment or are too common (*TP53* mutation occurs in >75% of tumors) their presence cannot inform prospective decision making for individual tumors [45]. Our initial work with ionizing radiation identified acute lactate perturbations as a biomarker of oxidative stress with excellent spatial and temporal resolution which can predict response as well as induced and intrinsic radio-resistance [27, 28]. In the current study, we demonstrate that acute lactate perturbations acts as a marker of Pt therapy response as well. Our data indicate that lactate levels correlate with Pt therapy dose and can differentiate between the CDDP and CBP derivatives, which is consistent with the differential cytotoxicity of these two Pt agents in HNSCC. Moreover, HNSCC cells which are resistant to CDDP and CBP, display a distinct profile with regard to acute lactate level changes, consistent with our previously described findings for ionizing radiation. Mutation of the *TP53* gene can exhibit profound effects on tumor cell metabolism through both loss of function (LOF) and gain of function (GOF) mechanisms, as shown previously by our group and others [35, 41, 48]. We have previously shown that loss of p53 function results in loss of metabolic flexibility which alters the ability of tumor cells to modulate oxidative stress, which may explain the differential response demonstrated in the current study [35, 41, 48].

Acute transient lactate perturbations act as a *sensitive* biomarker of Pt response. The *in vitro* experiments performed in this study demonstrated measurable lactate perturbations close to the GI50 values for the respective cell lines, which is in stark contrast to other *in vitro* assays (e.g., Western blotting) that often require much higher concentrations. Similarly, in the preclinical murine model, acute lactate perturbations corresponded with very small dose adjustments which generated comparatively subtle alterations in tumor growth delay. The sensitivity of this biomarker is one of the most encouraging aspects with regard to clinical translation. The mechanism linking acute changes in cellular lactate levels with genotoxic stress is still under active investigation by our group. Nevertheless, over the last 5 years, we have shown that intra-cellular lactate levels are susceptible to transient perturbations in the availability of reducing equivalents, specifically the NADH/NAD+ ratio. This ratio is perturbed not only by radiation-induced reactive oxygen species (ROS) as shown previously, but also by genotoxic stress generated by chemotherapeutic agents as shown here [27, 28, 36]. It is important to note that we are not making a statement regarding the overall importance of NADH/NAD+ in regulating cellular oxidative homeostasis, simply that it provides an potential mechanistic explanation for the measured changes in lactate. As shown above, intra-cellular changes in reducing equivalents are not isolated to NADH/NAD+ and can be measured in other reducing equivalents such as NADPH/NADP+. Since NAD+ and NADP+ exist in a relative equilibrium maintained by either direct or indirect metabolic mechanisms, genotoxic stress would be expected to generate global changes in cellular reducing potential [49–53].

Clinical translation of this preclinical biomarker in the case of solid tumors will require overcoming the challenges inherent in decreased tissue availability to directly assess lactate changes. Nevertheless, along with other investigators, we have shown that magnetic resonance spectroscopic imaging (MRSI) of ^13^C hyperpolarized (HP) lactate can generate the same information in a non-invasive manner [27, 36, 54–56]. HP-MRSI has been utilized by our group to demonstrate altered lactate metabolism in a murine model of AML [57]. Importantly, we have also shown that HP-MRSI can provide spatial resolution required to interrogate heterogeneous solid tumors [27, 56]. In hematopoietic malignancies, implementation of this metabolic interrogation approach would require timed blood draws and bone marrow biopsies following administration of chemotherapy. Although Pt agents are rarely utilized in the setting of hematopoietic malignancies, doxorubicin is part of the backbone of several widely utilized chemotherapy regimens, and is routinely employed in the treatment of leukemia [29–32]. Although the chemical structure, source and precise chemical mechanism of inducing DNA damage differs from Pt agents, free radical production contributes to its cytotoxicity [58–61]. Consistent with both Pt and IR, doxorubicin was shown to trigger a dose- and time-dependent decrease in cellular lactate levels in both HNSCC and AML cell lines.

This study, together with our previous publications, strongly suggests that metabolic interrogation can predict the effectiveness of DNA damaging agents such as ionizing radiation, Pt compounds and doxorubicin in multiple histologies. We do not expect that all potential chemotherapeutic regimens will register similar changes in cellular lactate levels, however a more unbiased and broad-based metabolomics approach may in fact identify similar biomarkers for most clinically employed agents. Together with ongoing efforts to predict clinical outcomes and tailor treatment regimens based on genomic and epigenetic profiling, metabolic interrogation may lead to development of truly individualized cancer treatments.

## MATERIALS AND METHODS

### Cells

Previously described cell lines (anaplastic thyroid carcinoma-ATC, head and neck squamous cell carcinoma-HNSCC) were obtained from an established cell line bank in the laboratory of Dr. Jeffrey N. Myers under approved institutional protocols. All cell lines were routinely tested and authenticated using short tandem repeat analysis [34]. Cells were maintained in either RPMI or DMEM growth media supplemented with glutamine, pyruvate, penicillin/streptomycin and 10% fetal bovine serum. HNSCC cell lines containing *TP53* constructs have been previously described by our group and are maintained in our laboratory [35, 36]. The acute myelogenous leukemia (AML) cell line, OCL-AML13, is a NPM-1 mutated cell line with wt-*TP53*; the cell line was obtained from the laboratory of Dr. Marina Y. Konopleva and cultured under standard conditions as previously described [37].

### Chemicals and antibodies

N-acetyl cysteine (NAC) were purchased from Sigma-Aldrich (St. Louis, MO). Carboplatin (CBP) (SAGENT Pharmaceuticals, Schaumburg, IL), cisplatin (CDDP) (Teva Pharmaceuticals, Sellersville, PA) and doxorubicin (Pfizer Inc, New York, NY) were obtained from the institutional inpatient pharmacy. The following antibodies were used in this study: anti-p53 DO-1(Santa Cruz Biotechnology, Santa Cruz, CA, USA), anti-phospho- P53 (Cell Signaling, Danvers, MA, USA), anti-p21 (EMD Millipore, Billerica, MA, USA), anti-PARP (Cell Signaling, Danvers, MA, USA), ATM (Santa Cruz Biotechnology, Santa Cruz, CA, USA), p-ATM (EMD Millipore, Billerica, MA, USA), p-Histone H2AX S139 (Cell signaling, Danvers, MA, USA), LDHa, LDHb (Abcam, Cambridge, MA, USA) and β-actin (Sigma, St. Louis, MO, USA).

### Metabolic studies

For lactate, NAD+/NADH and NADP+/NADPH measurements, cells were harvested at various time points following drug treatment using appropriate buffers and frozen in liquid nitrogen. Lactate, NAD+, NADH, NADP+ and NADPH levels were analyzed using commercially available colorimetric assays (BioVision, Milpitas, CA), according to the manufacturer's instructions.

### Cytotoxicity studies

Drug cytotoxicity was assayed using either clonogenic assays or the standard 3-(4,5-dimethylthiazol-2-yl)-2,5-diphenyltetrazolium bromide (MTT) assay. For clonogenic assays, cells were treated with the indicated drug. Fresh media were replaced and cells were incubated for colony formation for 10-14 days, then fixed and stained using a 0.05% crystal violet in 10% formalin solution. Colonies were counted and surviving fractions were determined based upon the plating efficiency of the control group. For GI50 measurements cells were seeded in 96-well plates and exposed to various drug concentrations. Drug effects were ascertained 72 hours later using a standard 3-(4,5-dimethylthiazol-2-yl)-2,5-diphenyltetrazolium bromide (MTT) assay as previously described [38]. Cell cycle analysis was performed using propidium iodide labeling of cells at various time points following exposure to drugs [39]. Drug GI50 values for the leukemia cell line were calculated using the commercially available CellTiter-Glo Luminescent Cell Viability Assay (G7572, Promega, Madison, WI).

### HNSCC tumors

Female athymic nude mice (8-12 weeks) (Envigo, Indianapolis, IN) were maintained in a pathogen-free facility and fed irradiated mouse chow and autoclaved, reverse osmosis treated water. The animal facility was approved by the American Association for the Accreditation of Laboratory Animal Care and met all current regulations and standards of the U.S. Department of Agriculture, U.S. Department of Health and Human Services and the National Institutes of Health. All procedures were approved by the Institutional Animal Care and Use Committee of The University of Texas MD Anderson Cancer Center. For flank tumors, cells (2×10^6^/mouse) were injected into both flanks of each animal. Tumor size was ascertained regularly throughout the experimental period using manual measurements as previously described [27, 36]. Tumors were allowed to grow for ~1 week prior to initiation of experiments. Following completion of experiments, tumors were harvested for either biochemical analysis or histologic and immunohistochemical analysis. The number of animals chosen for each *in vivo* experiment was based on previous experience with this animal model and the expected effect size. Statistical analysis of *in vivo* data was conducted as described below.

### Statistical analysis

All *in vitro* experiments were carried out at least in triplicate (for each condition) and were repeated to ensure reproducibility. All statistical analysis for *in vitro* experiments was conducted using two tailed, Student's t-test analysis with a cutoff p-value of 0.05 to demonstrate statistical significance. For all *in vivo* experiments statistical significance was determined using Student's t-test analysis with a cutoff p-value of 0.05 to demonstrate statistical significance.

## SUPPLEMENTARY MATERIALS FIGURES


