# Supplementary material for: Metabolic interrogation as a tool to optimize chemotherapeutic regimens
Source: Oncotarget. 2017 Feb 8;8(11):18154–65. doi: 10.18632/oncotarget.15186 (PMC5392315; doi:10.18632/oncotarget.15186)
Supplement: Supplementary file 1 [file oncotarget-08-18154-s001.pdf]

Metabolic interrogation as a tool to optimize chemotherapeutic regimens

SUPPLEMENTARY FIGURES

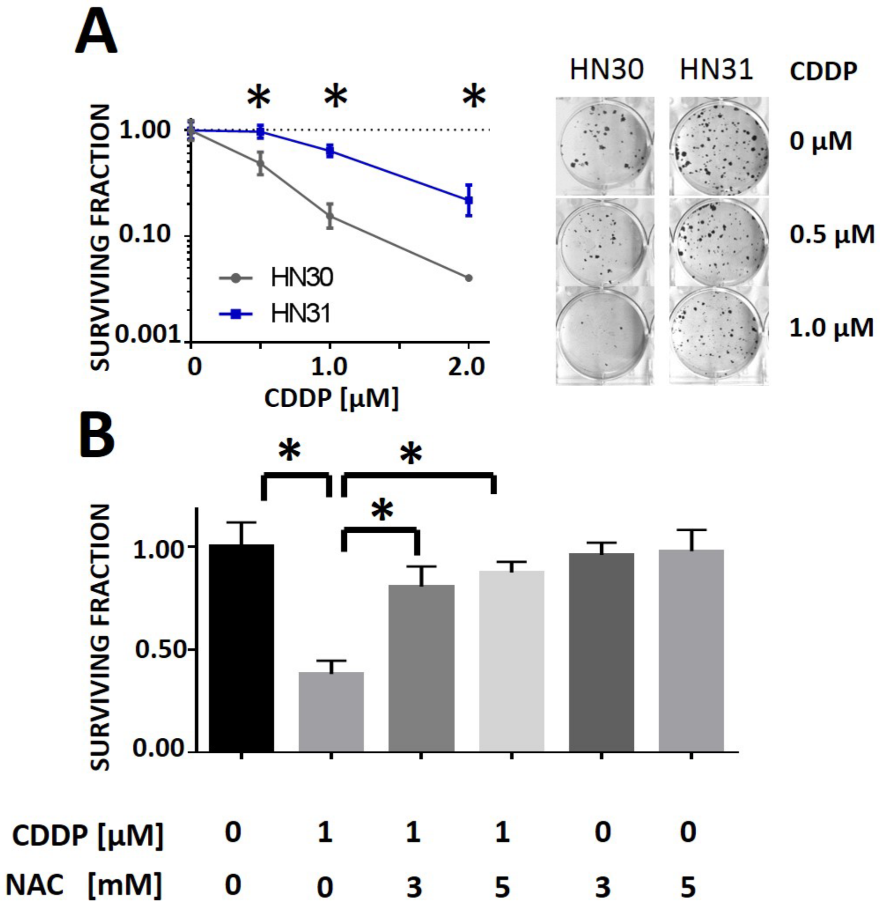

Supplementary Figure 1: HNSCC cells display differential CDDP sensitivity based on *TP53* mutational status. A. HN30 and HN31 cells were exposed to increasing CDDP concentrations. Differential effects on cell survival were ascertained using clonogenic survival assay. **B.** HN30 cells were exposed to CDDP in the presence of various concentrations of NAC. The effects of CDDP +/- NAC on cell survival were ascertained using clonogenic survival assays. * indicates p-value < 0.05 compared to corresponding control condition unless otherwise indicated. All values normalized to corresponding control condition. Each experiment was carried out at least in triplicate, with values indicating means and error bars representing standard deviation.

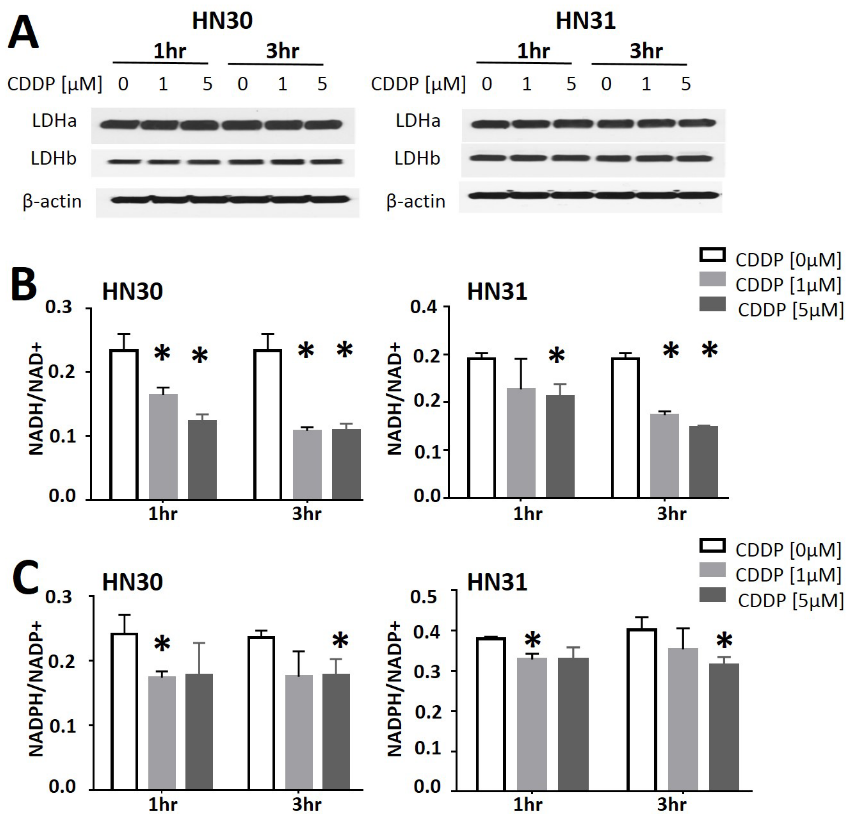

Supplementary Figure 2: Cisplatin perturbs cellular reducing equivalent levels. A. HN30 and HN31 cells were exposed to increasing CDDP concentrations for 1 or 3 hours. Cell lysates were probed for LDHa and LDHb expression. **B-C.** HN30 and HN31 cells were exposed to CDDP for 1 or 3 hours. Cell lysates were assessed for NAD+ and NADH (B) and NADP+ and NADPH (C) levels. * indicates p-value < 0.05 compared to corresponding control condition unless otherwise indicated. Each experiment was carried out at least in triplicate, with values indicating means and error bars representing standard deviation.

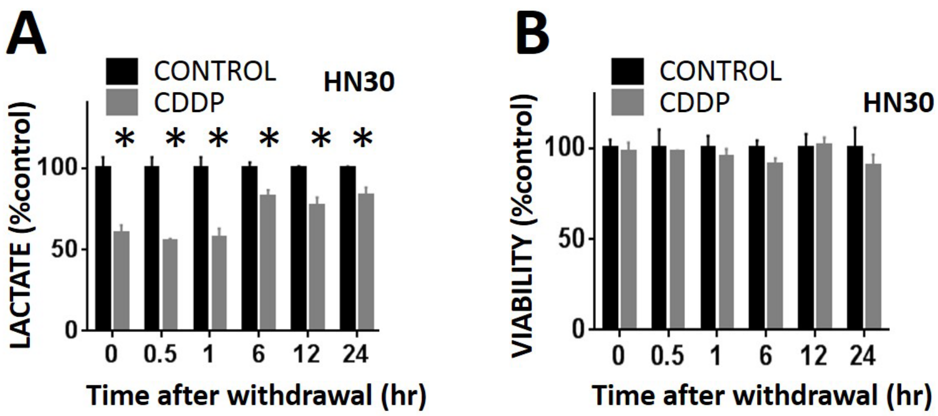

Supplementary Figure 3: Cisplatin effects on cellular lactate levels are transient. A. HNSCC (HN30) cells were exposed to CDDP [μM] for 1hr. CDDP was then removed and cells were harvested at various time points following withdrawal. Lactate levels were calculated at each time point and compared to the control (untreated) condition. **B.** Parallel cell cultures were subjected to MTT analysis to ascertain viability. * indicates p-value < 0.05 compared to corresponding control condition. All values normalized to corresponding control condition. Each experiment was carried out at least in triplicate, with values indicating means and error bars representing standard deviation.

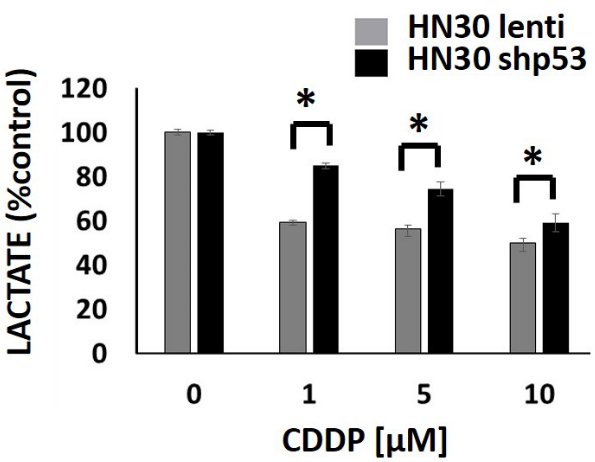

Supplementary Figure 4: Loss of wild type *TP53* blunts cisplatin effects on cellular lactate. CDDP [µM] induced a dose-dependent decrease in cellular lactate levels within 3 hr post exposure. HN30lenti cells demonstrated approximately 50% decrease in lactate levels compared to approximately 25% decrease in their counterpart, HN30shp53. * indicates p-value < 0.05 compared to corresponding control condition. All values normalized to corresponding control condition. Each experiment was carried out at least in triplicate, with values indicating means and error bars representing standard deviation.

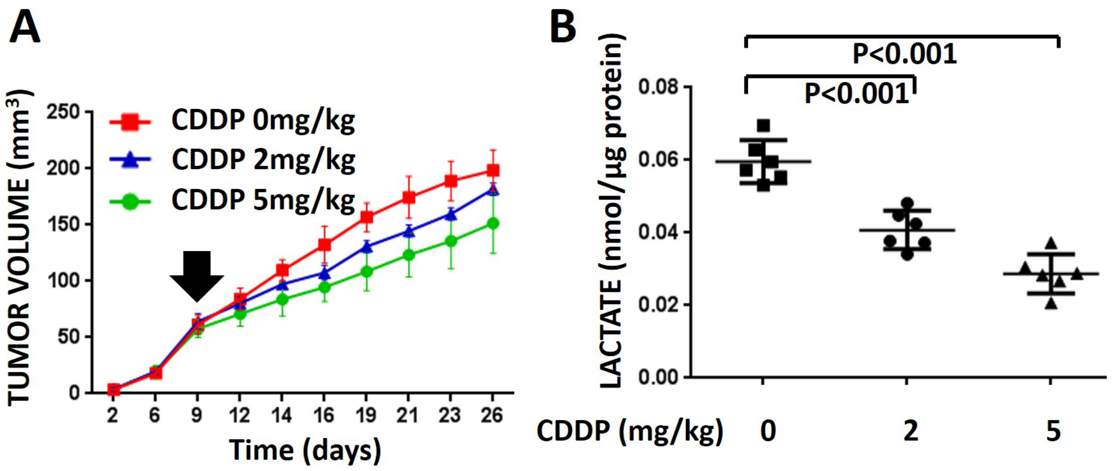

Supplementary Figure 5: Cisplatin-induced lactate perturbations correlate with effects on growth delay. A. Dual flank tumors generated from the HN31 cell line were exposed to a single dose of CDDP administered at day 9. One tumor (n=6/dose) was allowed to grow. Measurements of tumor size were obtained for the remainder of the experimental period and correlated to post-exposure lactate levels. Data are presented as mean volume with error bars indicating standard deviation. **B.** One tumor per animal (n=6/dose) was removed and analyzed biochemically for lactate levels at 3 hr post-exposure.

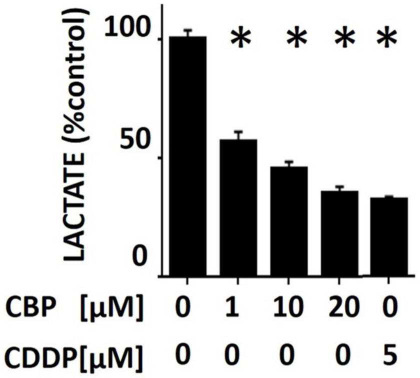

Supplementary Figure 6: Differential sensitivity of HNSCC cells to cisplatin (CDDP) and carboplatin (CBP) increases with time. HNSCC (HN30) cells were exposed to CBP or CDDP for 6 hr prior to measurements of cellular lactate levels. * indicates p-value < 0.05 compared to corresponding control condition. All values normalized to corresponding control condition. Each experiment was carried out at least in triplicate, with values indicating means and error bars representing standard deviation.

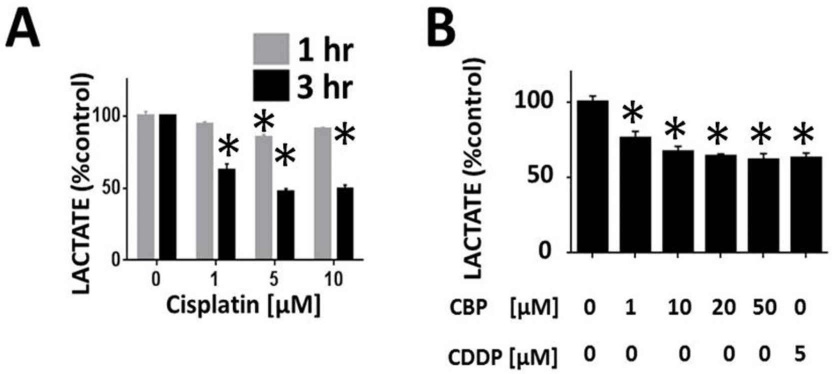

Supplementary Figure 7: CDDP and CBP induce changes in cellular lactate levels in anaplastic thyroid carcinoma. ATC cells (U-HTH83) were exposed to the indicated doses of CDDP for 1 or 3 hr (A) or CDDP or CBP for 1 hr (B). Cellular lactate levels were measured and compared to control condition. * indicates p-value < 0.05 compared to corresponding control condition. All values normalized to corresponding control condition. Each experiment was carried out at least in triplicate, with values indicating means and error bars representing standard deviation.
